# A simplified non-greenhouse hydroponic system for small-scale soilless urban vegetable farming

**DOI:** 10.1016/j.mex.2022.101882

**Published:** 2022-10-09

**Authors:** Margaret S. Gumisiriza, Patrick A. Ndakidemi, Ernest R. Mbega

**Affiliations:** School of Life Sciences and Bio-engineering, The Nelson Mandela African Institution of Science and Technology, P.O Box 447, Arusha, Tanzania

**Keywords:** Small-scale urban agriculture, Vegetable production, Kratky hydroponics, Soilless farming, Sustainable cities

## Abstract

Majority of under-developed countries continue to face a challenge of food insecurity around urban areas resulting from factors such as; limited access to arable land. This study aimed at developing a simplified low-tech hydroponic system for growing leafy vegetables alongside testing its economic viability. This was intended to support urban vegetable production and henceforth contributing to food security more so in under-developed states dealing with the challenge of increasing urban population vs. reducing arable land around urban/ peri-urban areas. A hydroponic unit for growing 60 leafy vegetables (using lettuce as a study crop) under non-controlled environmental conditions was designed and developed using low-cost and low-tech materials. Kratky hydroponic method which involves growing crops using water as a media without the need for water pumps and electricity was used. A study was also carried out to assess the profitability of the system. The results indicated a: net present values of 16.37$, internal rate of return of 12.57%, profitability index of 1.1 and non-discounted payback period of approximately 8 months (4 cropping seasons).

These findings showed that the system has the potential to improve urban food production and availability in especially in developing countries in a profitable manner. Vegetable production using the hydroponic system can also contribute to:

• tachievement of sustainable development goals, 2 (zero hunger) and 3 (good health and wellbeing);
• improvement in urban agriculture production and income generation among urban farmers;
• enhanced adoption of low-cost, low-tech, environmental-friendly and sustainable farming systems.

## Graphical abstract

**Figure:**
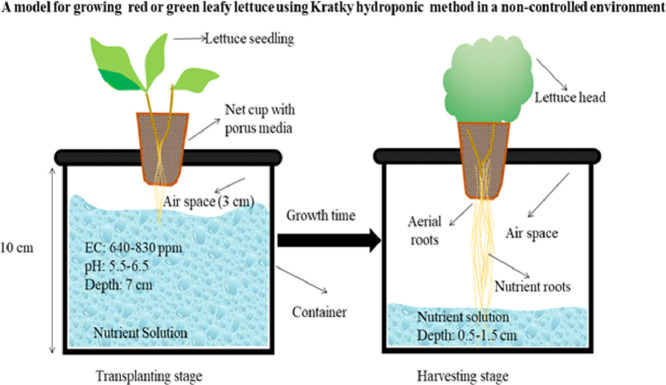

Specifications table

**Table:** 

Subject Area:	Agricultural and Biological Sciences
More specific subject area:	Hydroponic farming; urban agriculture
Method name:	Kratky hydroponics system
Name and reference of original method:	Name: Kratky method - hydroponic farming Kratky B.A. [1]. Growing direct-seeded watercress by two non-circulating hydroponic methods. Vegetable crops, VC-7.
Resource availability:	The main data (with methodology besides study results) and resource articles are at; • https://doi.org/10.1016/j.scs.2022.103923 • https://www.ctahr.hawaii.edu/hawaii/downloads/A_Suspended_Pot_Non-circulating_Hydroponic_method.pdf

## Introduction and study rationale

The growing population rates across the globe especially in developing states coupled with the decreasing arable land have intensified the demand for food prompting interventions for fresh and sustainable farming practices [2]. Moreover, the impacts from climate change have also increased recognition of crop modeling methods in particular [3]. This is due to the fact that these have heavily impacted on food security in majority of the countries mostly under-developed states. There is already a move from traditional farming systems to protected farming systems mainly soilless farming [4]. Fresh farming systems to produce vegetables specifically while reducing land use are being exploited [5]. Some studies have already shown soilless culture to be an alternative cropping system to traditional farming in terms of reducing the problems related to conventional farming such as; low profitability [6]. Organisations like: Food and Agriculture Organisation (FAO) have advocated for adoption of simplified soilless systems in developing countries to improve food security [7].

Unfortunately, adoption and implementation of these soilless systems in majority of African states for instance is still limited due to lack of adequate knowledge about the technology and the high initial costs associated with it [8, 9]. The purpose of this study was to develop a simplified low-cost vertical hydroponic unit to increase vegetable production within urban centers in developing states that still have low adoption of soilless farming technologies and yet battling with food insecurity challenges. Soilless farming is a sustainable method for supporting vertical agriculture in urban areas [5]. Vertical farming in itself has the capacity to produce food in a climate-resilient way while using low land as compared to traditional soil culture systems. The farming method can support production and availability of nutritious fresh food especially in and around highly urban/semi-urban populated areas [10]. The Kratky hydroponic system was adopted for the study under non-controlled environmental settings in a vertical manner at a small scale. A cost-effective analysis study was carried out on the same experiment [11] which showed that the system has the potential to produce vegetables in a profitable way.

## Background to Kratky hydroponic method

**Figure 1:**
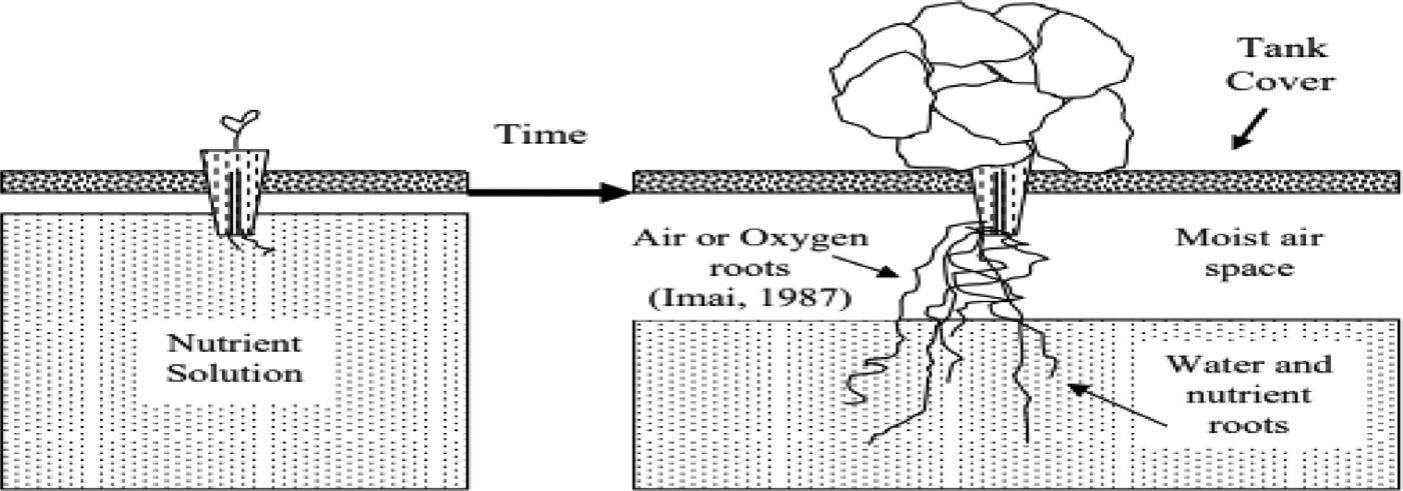
A representation of a non-circulating hydroponic system using suspended net cup. Source: [12]

This is a passive hydroponic method where plants are suspended above a nutrient rich solution which is filled in a container at once prior to transplanting [12]. The plants are automatically watered since the entire media is spontaneously moistened by capillary action. The nutrient solution reduces as the plant grows thus creating an increasing aeration space (Fig. 1). The method is slightly similar to DWC (Deep Water culture) hydroponic method as they both don't require refilling of the nutrient solution during the growth cycle. The difference between the two methods is that with Kratky, the pots must be exposed to air at least 50% since it does not use air pumps for aeration. Kratky hydroponic method was named after Bernard Kratky who invented it [13]. It favors mainly growth of small crops like; vegetables (lettuce) and herbs which have a fast rate of growth as these small crops can be grown with one initial application of the nutrient solution for the entire cropping period [14]. The extra costs incurred in other hydroponic methods are eliminated as the Kratky method does not necessitate the use of timers, air pumps, climate monitoring systems, or additional labour [12].

In a nut shell, Kratky hydroponics operates under four major principles [15], which are:

i) air roots should have relatively high moisture and exposed to air;
ii) roots must not run out air;
iii) nutrient roots are responsible for nutrient and water uptake;
iv) nutrient solution level should not be increased but can remain the same or be decreased.

## Study site

The experiment was carried out in 2021 around Wakiso district located in central Uganda. This district is close to the country's’ capital city (Kampala). Uganda is situated in the Eastern part of Africa and receives an average rainfall amount of 1500-2000 mm annually. The country had a 5.2% population rise in 2021 with a total number of approximately 44 million people majority of whom were living in and around urban areas.

## Structural design for non-greenhouse hydroponic system for lettuce cultivation

**Figure 2:**
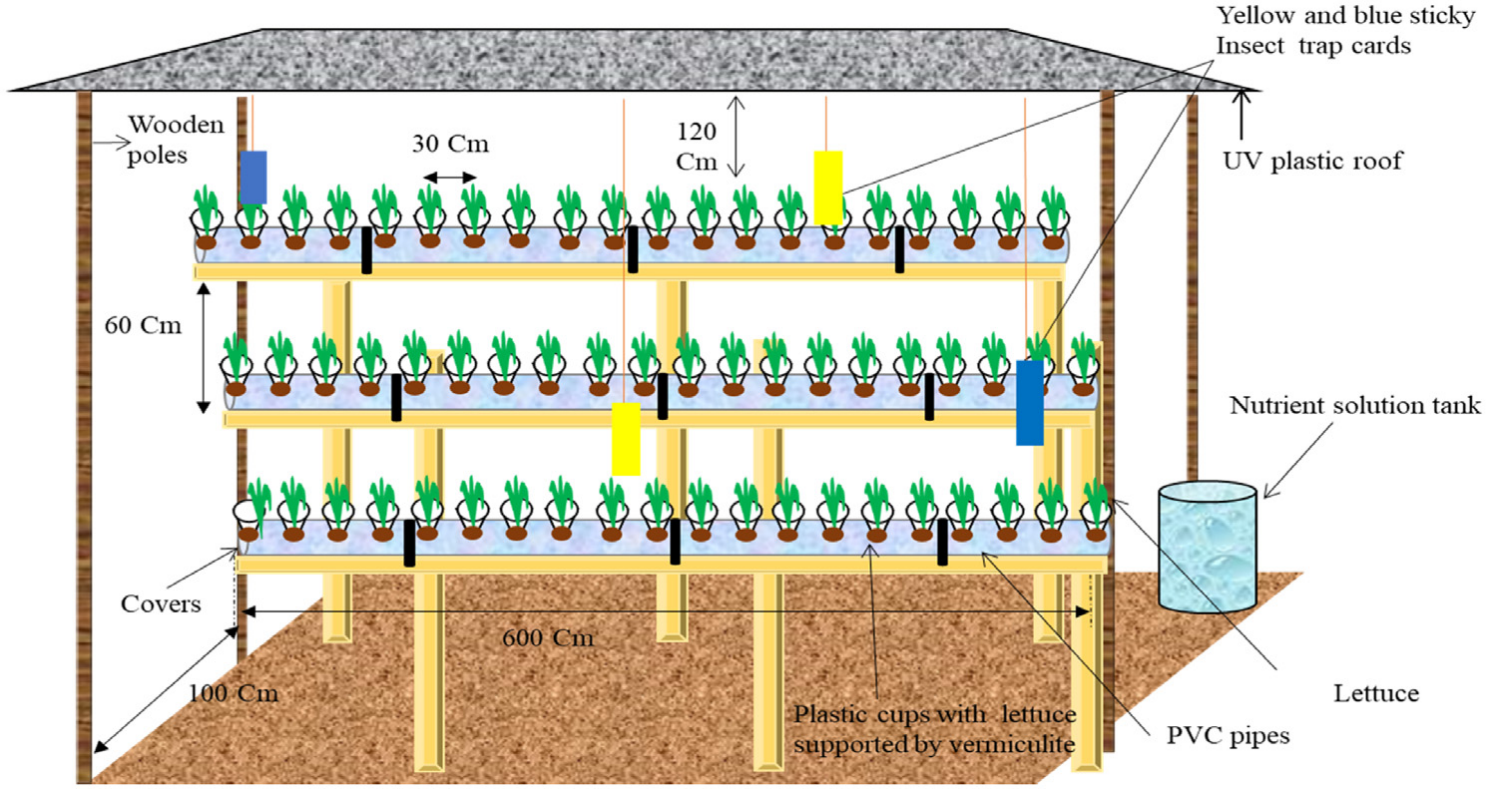
An image illustrating the design of a 6 × 1metres vertical hydroponic unit for lettuce cultivation outside the green house.

A low-tech hydroponic system for growing 60 heads of lettuce using Poly Vinyl Chloride (PVC) pipes and Kratky hydroponic method without a greenhouse was designed (Fig. 2). A 3-layered vertical wooden rack (600 cm in length) with a spacing of 60 cm between levels and total height of 300 cm from the bottom to top was constructed. The rack occupied a space of 6*1 meters. An Ultraviolet (UV) plastic polythene roof to provide shade from direct sunlight and rainfall was built on top of the rack at 120 cm above the last level on top. A 600 cm (20 feet) Poly vinyl chloride (PVC) pipe with 20 holes (30 cm apart) drilled on it was assembled on each level of the rack.

The hydroponic rack also had 2 blue and 2 yellow insect trap-cards fixed and hanging from the UV-plastic roof. These were fixed once during the growing cycle with an aim of combating pests such as; leaf miner and white flies known for sucking out liquid from leafy vegetables. The Polyfeed (19:19:19) crystalline water soluble fertilizer was thoroughly mixed with 90 litres of water (10 grams/litre) in a small tank. The nutrient solution was then filled in the PVC pipes with 30 litres per pipe.

A Total Dissolved Solutions (TDS) meter reader was used to measure and monitor the potential of hydrogen (pH) and Electrical Conductivity (EC) in the nutrient solution. EC gives an insight into the amount of nutrients available in the solution while pH is an indicator of the acidity or alkalinity in the solution which determines nutrient uptake. Average pH was 5.9 while average EC was 675 parts per million (ppm). 3-week old lettuce seedlings were placed into the regular small white disposable cups (Fig. 4 right hand side) with holes drilled around them to allow nutrient uptake through the roots.

These seedlings in the cup were supported by saw dust mixed with small gravel stones (less than 1 cm in diameter). This mixture was made in a 1:1 ratio to balance the water retention and drainage.

An aeration space (approximately 2-3 cm) was left between the bottom of the cup and the top level of the nutrient solution in the PVC pipe to further improve oxygen supply to the roots and solution. Small holes (2 cm) were also drilled at the two ends of each pipe to support inflow of air into the nutrient solution. The cups with plants were then fitted onto the PVC pipe holes using a completely randomized design.

The step wise process of construction and assembling of the system involved:

i) construction of the wooden rack;
ii) fitting of the UV plastic rack on top of the wooden rack;
iii) drilling holes onto the PVC pipes and disposable cups as well as mixing the media;
iv) fitting of the PVC pipes on to the different levels of the rack;
v) mixing the nutrient solution in the small tank and later filling it in the PVC pipes;
vi) transplanting the lettuce seedlings into the cups with support of the media.

The process requires a carpenter who is responsible for constructing the wooden rack and fitting it with the UV-plastic in a day with assistance of a casual laborer to offer support in fitting the system and the necessary equipment. One casual laborer/ farmer is needed to prepare seedlings, transplant and take care of the plants during the growth stages till harvest as no need of fertilizer addition is required. The planting of the seeds is made a month before the transplanting time or anticipated harvesting time of the cropping cycle. The cropping season for the lettuce plants was articulated at 2 months between transplanting and first harvest. Monitoring the plants for any changes caused by factors that may affect their growth such as: pests, or environmental related conditions is necessary.

**Figure 3:**
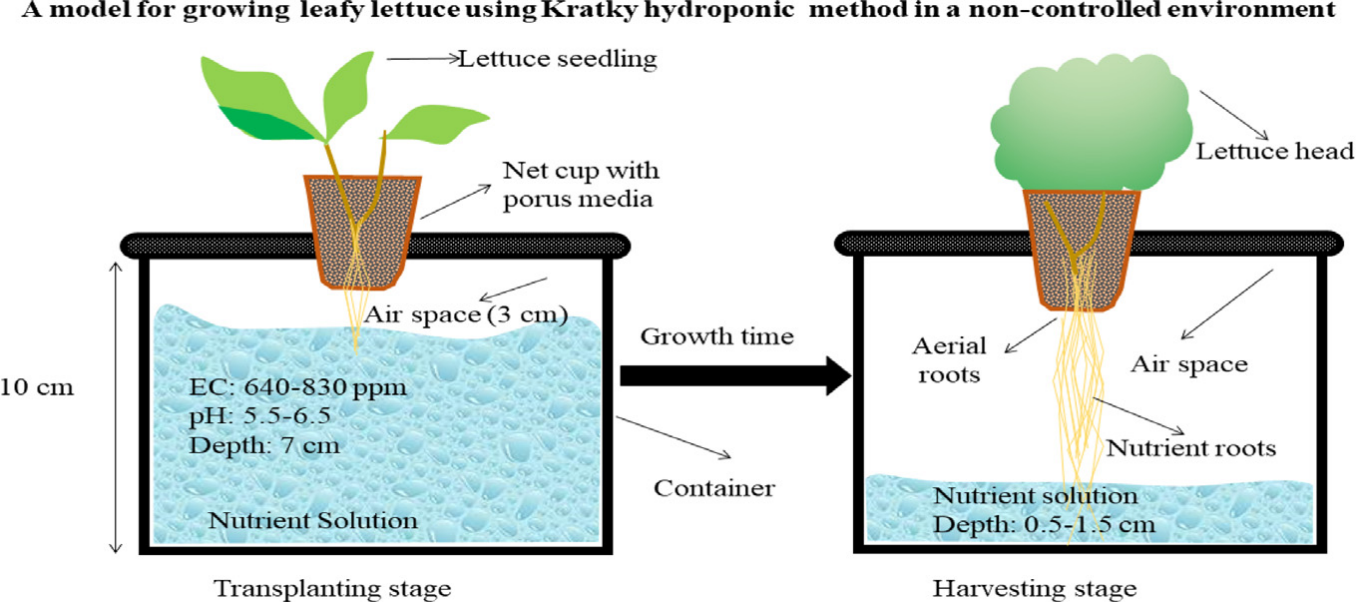
A model for growing leafy lettuce using Kratky hydroponics method under open field conditions.

**Figure 4:**
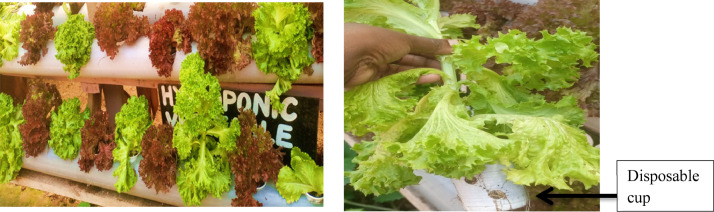
Hydroponic lettuce production using Kratky method outside the green house (left hand side) and hydroponic lettuce inside the white disposable cup (right hand side).

Source: [11].

Fig. 3 illustrates the detailed characteristics or necessary conditions for lettuce cultivation using Kratky hydroponic system (with PVC pipes) outside the green house while Fig. 4 shows the actual lettuce cultivation (experiment) using the system in an urban home setting and lettuce plant.

Table 1 on the other hand shows the detailed raw materials, quantities and associated costs required for construction of the hydroponic unit.

**Table 1:** Annual production costs for a small scale vertical hydroponic unit for lettuce production outside the greenhouse.

	Item	Quantity	Unit cost ($)	Total cost ($)
	**Fixed costs**			
1.	Construction of wooden rack with UV plastic	1	52	52
2.	PVC pipes	3	7	21
3.	PVC pipe covers	6 covers	1	6
4.	Water tank	1 (100 litres)	9	9
5.	TDS meter reader	1	10	10
	**Variable costs**			
6.	Water	1 unit	2	2
7.	Lettuce seeds (2gm/sack)	3 sacks (2gm/sack)	0.4	1.2
8.	Poly feed fertilizer	6kgs	4.4	26.4
9.	Insect sticky traps	1 packet (6 pieces)	7	7
10.	Disposable cups	6 dozens	0.5	3
11.	pH control kit	1	14	14
12.	Seedlings grow tray	1tray (150 seeds)	4	4
13.	**Sundry costs**		15.1	15.1
	**Total**			**170.7**

Source: [11].

Similar number of lettuce heads were cultivated using conventional farming (using soil filled in black potting bags) and the total cost of production was 129.2$. This was lower than that of the hydroponic system as anticipated due to the less number of raw materials required. However the same farming system (conventional method) required more space (8 × 2 meters) and water (103 litres) for irrigation as compared to the hydroponic farming system (90 litres) per cropping cycle.

## Study limitations and recommendations

Inconsistence/slow rate in the growth and maturity of the vegetables was observed during the experimental study. More research can be carried out to isolate likely causes of this such as; nutrient uptake, diseases and/or environmental conditions (such as: high/low temperatures) alongside identifying subsequent solutions to the challenge to improve on the performance of the system.

## Conclusion

The results showed that non-greenhouse Kratky hydroponic method is cost effective and can be used to to support and increase food production around urban or semi-urban areas facing challenges of limited arable land and increasing food demand for the growing population. This vertical hydroponic system is suitable for vegetable production in partial or full shade small spaces around urban or semi-areas such as: home steads, schools, offices and apartments. The system can be fitted on verandas, balconies, front or backyard gardens etc… It supports low-cost hydroponic vegetable production through eliminating production costs related to greenhouse construction, timers, aerators and climate monitoring systems. It also eliminates the complexity surrounding the high technology related with other hydroponic farming methods. This makes it easier for farmers in low developed states such as; those in Africa who cannot afford the high financial costs associated with green house hydroponic crop production to adopt.

## Declaration of competing interest

The authors declare that they have no known competing financial interests or personal relationships that could have appeared to influence the work reported in this paper.

## Data Availability

The link for the co-submission has been submitted
